# Treatment of Nail Psoriasis: Common Concepts and New Trends

**DOI:** 10.1155/2013/180496

**Published:** 2013-05-13

**Authors:** Yasemin Oram, A. Deniz Akkaya

**Affiliations:** Department of Dermatology, V.K. Foundation American Hospital of Istanbul, Turkey

## Abstract

The lifetime incidence of nail involvement in psoriatic patients is estimated to be 80–90%, and the nails can be affected in 10% to 55% of psoriatic patients. Psoriasis may also solely involve the nails, without any other skin findings, in which the treatment can be more challenging. Nail psoriasis may lead to considerable impairment in quality of life due to aesthetic concerns and more importantly limitations in daily activities resulting from the associated pain, which may be overlooked by the physicians. Several topical and systemic treatment modalities, as well as radiation and light systems, have been used in the treatment of nail psoriasis. In the last decade, the introduction of biologic agents and the utilization of laser systems have brought a new insight into the treatment of nail psoriasis. This paper focuses on the recent advances, as well as the conventional methods, in treating nail psoriasis in adults and children, in reference to an extensive literature search.

## 1. Introduction

Psoriasis is a chronic skin disease that causes significant distress and morbidity. Although the skin manifestations are more characteristic, the lifetime incidence of nail involvement in psoriatic patients is estimated to be 80–90%, and the nails can be affected in 10% to 55% of psoriatic patients [1–3]. Moreover, psoriasis may involve the nails only, without any other signs of skin findings [1, 4]. Nail psoriasis has been shown to be associated with longer duration of skin lesions. There is an association between the duration of psoriasis and the severity of nail involvement [2, 3, 5]. Nail psoriasis is also associated with higher disease severity [3, 6]. However, it may also occur in 40% of patients with mild psoriasis [2]. It is slightly more common in male patients than females [3, 6]. Nail psoriasis leads to considerable impairment in quality of life due to aesthetic concerns and more importantly limitations in daily activities resulting from the associated pain [2, 7].

Nail psoriasis may show different clinical presentations according to the structure that is involved within the nail unit. Nail matrix involvement leads to irregular nail pitting (the most common finding of nail psoriasis), dystrophy, and leukonychia; nail bed involvement causes onycholysis, subungual hyperkeratosis, splinter hemorrhages, oil drop patches, and nail thickening, whereas nail fold involvement may result in paronychia [1, 8, 9]. In cases of very severe inflammation, combined nail matrix and nail bed psoriasis may develop, forming “psoriatic crumbly nail.” Psoriatic nail constitutes a risk factor for secondary mycotic infections, which can occur in up to 27% of the cases. This coexistence should be excluded by mycologic examination before treatment [10].

There is striking association between nail psoriasis and a high risk of psoriatic arthritis, a chronic inflammatory arthropathy, with a prevalence of nail involvement among patients with psoriatic arthritis as high as 70%. Nail involvement may precede arthritis or may be considered as a predictor of future psoriatic joint damage [3, 11]. Nail bed involvement prevalence has been found higher in patients with psoriatic arthritis [11]. Psoriatic arthritis mainly involves distal interphalangeal joints and is characterized by dactylitis, enthesitis, osteolysis, and periarticular new bone formation [12]. A possible explanation for this association might be the close anatomical link between the nail unit and the distal interphalangeal joint. Inflammation of the extensor tendon enthesis, which are the attachment points of ligaments, tendons, and joint capsules to bone, can extend to the nail unit and result in psoriatic nail changes [8].

The Nail Psoriasis Severity Index (NAPSI) has been developed as an objective and reproducible tool, which helps to estimate the nail involvement and therefore to standardize the treatment outcome assessments. The nail is divided into quadrants, based on the signs of involvement of the nail matrix and the nail bed, rated with 0 or 1 [13]. NAPSI scoring system has some limitations, as it does not specifically consider the severity of nail matrix or nail bed involvement. In a recent report by Mukai et al. [14], NAPSI scores were evaluated in psoriatic patients using acitretin, and it was concluded that the method was easy and rapid in measuring the improvement of the treatment; however, it did not quantify the existing lesions and might not detect small changes. In 2007, a modified NAPSI (mNAPSI) was proposed by Cassell et al. [15] in order to increase the sensitivity of NAPSI, using a qualitative gradation of severity for each parameter from 0 to 3 in each quadrant. Although it can be time consuming and impractical for the clinicians in an outpatient clinic, mNAPSI demonstrates excellent interrater reliability and validity in the assessment of psoriatic nail disease [8, 15]. Digital photography might be an easy and convenient method for monitoring the progression of the nail involvement during the treatment [8].

Although there exist several treatment options for psoriasis, there are no existing guidelines or consistent treatment algorithms for the treatment of nail psoriasis, as the amount of published evidence available is limited. Recently, a systematic review evaluating the randomized controlled trials has provided some evidence concerning the management of nail psoriasis [16]. The management of nail psoriasis has been challenging particularly when the nail involvement is the only manifestation of the disease. Low penetration of the topical medications and slow growth rate of the nails are the main factors for this difficulty. Moreover, most of the therapies require prolonged treatment and continuity, sometimes with side effects and/or disappointing results. Obviously, it may have a negative impact on patient's compliance, motivation, and quality of life [4].

## 2. General Nail Care

The Koebner, or isomorphic response, which is the formation of new psoriatic lesions at sites of physical injury to the skin, is a well-known phenomenon. Psoriatic changes of the nail unit can also be triggered by minor traumas such as manicure, biting the nails, picking or trimming the cuticle, clearing subungual debris, or wearing tight-fitting shoes. An important part of the treatment is to ensure that the patient is avoiding all the factors that may exacerbate the disease. The physical trauma should be discouraged, the hands and feet should be thoroughly wiped dry, and the nails must be kept short [8, 9].

## 3. Topical Therapy

There is limited evidence for the efficacy of topical therapies in nail psoriasis. Various topical treatment modalities have been used in nail psoriasis including corticosteroids, dithranol, fluorouracil, vitamin D_3_ analogues, tacrolimus, cyclosporine, and tazarotene [1, 4, 7, 8] (Table 1).

The main topical treatments for nail psoriasis have traditionally comprised potent corticosteroids applied under occlusion. The clobetasol propionate at a concentration of 0.05% in cream or gel vehicle has been the most recommended topical treatment. However, they may cause atrophy, depigmentation, telangiectasia, and bone reabsorption. In 1999, Baran and Tosti [29] first described the use of 8% clobetasol propionate in nail lacquer vehicle in the treatment of 45 patients with nail psoriasis. The nail lacquer is a solution that helps to prevent the adverse effects caused by the use of intralesional corticosteroids or corticosteroids in cream or gel applied to the skin. Consequently favorable results have been observed with 8% clobetasol propionate lacquer that has effective transungual penetration and therapeutic effect both on nail bed and matrix lesions, while lacking side effects [17, 23].

Dithranol is an anthracene derivative with antiinflammatory and antiproliferative actions [8]. In a prospective study, dithranol (anthralin) ointment with a 0.4–2% concentration has been shown to be effective in 60% of 20 patients particularly in the treatment of onycholysis and subungual hyperkeratosis using daily short-contact therapy. The ointment was applied for 30 minutes daily for 5 months. Side effects included local irritation and temporary staining of the nail [31].

Fluorouracil is a pyrimidine analog, which inhibits the thymidylate synthase. Topical 5-fluorouracil (5-FU) in 1% or 5% concentrations in different vehicles has been used in nail psoriasis with unpredictable results. In a prospective study, the application of 1% fluorouracil solution twice daily for 6 months demonstrated marked improvement in nail pitting and hyperkeratosis in 85% of patients [32]. However, another small double-blinded study failed to show any benefits from topical 5-FU lotion 1%, combined with urea and propylene glycol. Besides, onycholysis was aggravated with the treatment [28]. The reported side effects such as pain, infection, nail loss, hyperpigmentation, onycholysis, and skin irritation, in the small number of studies conducted, are the reasons to limit its use.

Vitamin D_3_ analogues of calcipotriol, calcitriol, and tacalcitol have been used in the treatment of nail psoriasis by inhibiting keratinocyte growth and differentiation and suppressing T-cell activity and cytokine production [1, 7, 21, 33]. In a double-blind study involving 58 patients with nail bed psoriasis, topical application of calcipotriol ointment twice daily was found to be as effective as twice daily application of betamethasone dipropionate in reducing subungual hyperkeratosis after 3–9 months [30]. Side effects include periungual irritation, erythema, and burning [7]. Combination therapy of topical corticosteroids with vitamin D_3_ analogues including calcipotriol and tacalcitol has been shown to be effective, although there is no standard therapeutic regimen. The antiinflammatory effect of corticosteroids together with the antiproliferative and immunomodulatory effects of vitamin D_3_ not only enhances the outcome of the treatment but also reduces the risk of adverse effects [8]. Rigopoulos et al. [26] demonstrated that the combined treatment with calcipotriol cream (once daily, 5 days a week) and clobetasol propionate cream (once daily, 2 days a week) for 6 months resulted in a 72% reduction at subungual hyperkeratosis. The synergistic effect of topical vitamin D_3_, which mainly affects the nail bed, togetherwith topical corticosteroid that is more effective on nail matrix, may provide safer and better treatment for nail psoriasis [20, 21, 30].

Tazarotene is a synthetic retinoid derived from vitamin A that downmodulates keratinocyte hyperproliferation, differentiation, and inflammation. Studies using tazarotene gel 0.1% have been conducted with successful results in nail psoriasis [25, 27]. Rigopoulos et al. [22] reported that tazarotene 0.1% cream was as effective as clobetasol propionate 0.05% cream in improving onycholysis, discoloration, pitting, and hyperkeratosis after 12 weeks in a double-blind study of 46 patients. In a small study, the efficacy and safety profile of tazarotene 0.1% hydrophilic ointment under occlusion was evaluated in the treatment of nail psoriasis of 6 patients. Clinically significant improvement in nail involvement, especially the subungual hyperkeratosis and onycholysis, was observed after 6 months of treatment [18]. Topical tazarotene has been generally well tolerated with mild erythema, local irritation, desquamation, and burning [25]. The combination of tazarotene with a topical corticosteroid might be helpful in decreasing the irritation caused by tazarotene while achieving an enhanced effect with two different molecules [7].

Several studies have shown that a topical calcineurin inhibitor, tacrolimus ointment, may penetrate the periungual skin and can be used to treat nail dystrophy caused by lichen planus or chronic paronychia [7, 34]. Tacrolimus in 0.1% and 0.03% ointment was found to be effective in psoriasis due to its immunosuppressive property. De Simone et al. [19] have reported good clinical results in nail psoriasis with topical tacrolimus 0.1% ointment application after 12 weeks. It seemed to be equally effective on nail bed and matrix lesions without having severe side effects.

Topical cyclosporine preparation showed to improve pitting and onycholysis in a small, placebo-controlled study. Oil-dissolved 70% solution of cyclosporine has been used in 8 patients for 12 weeks, and complete resolution or substantial improvement has been reported in all patients [24]. However, topical cyclosporine is not as effective as the systemic form in nail psoriasis, and further studies are necessary to optimize the vehicle and stability of the topical administration [35].

## 4. Intralesional Therapy

Despite a relative paucity of controlled studies, intralesional injections with corticosteroids are considered to be a standard treatment for nail psoriasis [1]. Triamcinolone acetonide is the most widely used agent in doses of 2.5–10 mg/mL at up to four injection sites (two into the proximal nail fold and two in the lateral nail fold), used bimonthly for 5-6 months. Injections to the proximal nail fold with 28- and 29-gauge needle syringes or with needle-less injectors are very effective in treating nail matrix disease such as pitting or ridging [1, 9, 36]. Up to 70–90% of psoriatic patients with both nail matrix and nail bed lesions respond to intralesional steroids, except for onycholysis, which shows a less pronounced response. Corticosteroid injections can cause considerable pain, atrophy, despigmentation, secondary infection, inclusion cysts, subungual hemorrhage, and tendon rupture [9, 36]. This treatment requires repeated injections.

Saricaoglu et al. [37] reported a case of severe psoriatic nail disease successfully treated with intralesional methotrexate at a dose of 2.5 mg of weekly injections for 6 weeks. The significant improvement in subungual hyperkeratosis and pitting was maintained after 2 years of treatment. The most important limitation of the therapy was the severe pain during injections.

## 5. Phototherapy, Radiation Therapy, and Laser Therapy

Although phototherapy with narrow-band UVB and photochemotherapy with UVA, in addition to oral psoralen (PUVA), have been successfully used in psoriasis, there is not enough evidence to support their beneficial effects in nail psoriasis. Several small studies have shown that PUVA can help psoriatic nails with variable results. The pitting and onycholysis have demonstrated poor response to PUVA treatment [38]. A recent study on the penetration of UV lights in normal human cadaveric fingernail plate showed that the nail plate completely blocks UVB light but only a minimal amount of UVA penetrates the nail [39]. This may explain the limited effect of PUVA in nail psoriasis.

Superficial radiotherapy, electron beam therapy, and Grenz rays have been infrequently used in the treatment of nail psoriasis with temporary benefits. The possibility of localized fibrosis and carcinogenesis should be considered in radiation therapy [7, 8]. Soft X-rays have been used for very thick psoriatic nails in one case, in fractionated doses of 1.5 Gy for a total of 13.5 Gy (43 kV, 25 mA, 0.6 mm aluminum filter) at 1- and 2-week intervals. The nail plates became normal after 12 months of therapy [40]. 

In recent years photodynamic therapy (PDT) and pulsed dye laser (PDL) have been described for the treatment of nail psoriasis regarding their proven effects on plaque type psoriasis. We have used 595 nm PDL (7 mm spot size, 1,5 ms pulse duration, 8–10 j/cm^2^ energy) in nail psoriasis of 5 patients, once monthly for 3 months, with significant improvement, particularly of nail bed lesions [41]. Mean NAPSI scores declined from 21.2 at baseline to 3 at 1 month after 3 sessions. Treewittayapoom et al. [42] found PDL to be effective in both nail bed and matrix lesions, in a left-to-right comparison study of 20 patients, evaluating the effect of two different pulse durations (6 ms, 9 j/cm^2^ versus 0.45 ms, 6 j/cm^2^ both with 7 mm spot size). A significant reduction in NAPSI scores from baseline was observed in both groups after 6 months of the first treatment, while no significant difference was found between the longer and the shorter pulse duration groups. Fernández-Guarino et al. [43] evaluated the efficacy of PDT and PDL in the treatment of nail psoriasis in a left-to-right comparison study of 14 patients. Both hands were treated with 595 nm PDL (7 mm, 6 ms, 9 j/cm^2^), following 3 hours occlusion of methyl-aminolaevulinic acid (MAL) to one hand, once a month for 6 months. After 3 hours occlusion of methyl-aminolaevulinic acid (MAL) to one hand, both hands were treated with 595 nm PDL (7 mm, 6 ms, 9 j/cm^2^), once a month for 6 months. They showed that both treatments were equally effective in nail bed and nail matrix lesions; hence MAL did not play any role in the improvement of nail psoriasis.

## 6. Systemic Therapy

Systemic treatment can be recommended in severe localized nail psoriasis when topical or intralesional therapy has failed or in the treatment of moderate-to-severe psoriasis vulgaris accompanied with nail involvement [4, 7, 9] (Table 2).

Retinoids are vitamin A analogues that influence epidermal differentiation, proliferation, and immunomodulation. Etretinate and its active metabolite acitretin have been used in psoriasis. Tosti et al. [46] showed a 41% mean improvement in NAPSI scores after 6 months of treatment of low-dose acitretin (0.2–0.3 mg/kg/day) in nail psoriasis. Ricceri et al. [50] reported a case with severe nail psoriasis that showed marked improvement in 2 months of therapy of acitretin at a dose of 0.5 mg/kg, combined with urea nail lacquer. Acitretin may decrease the thickness of the nail resulting in nail atrophy and fragility. Therefore patients with thickened nails and severe subungual hyperkeratosis are better candidates for acitretin treatment [8]. As retinoids are teratogenic and have been associated with significant hepatotoxicity and hypertriglyceridemia, they should be reserved for very resistant and severe nail involvement [7].

Although the efficacy of methotrexate and cyclosporine on plaque type psoriasis has been reported previously, the literature consists of few publications regarding the efficacy of the two treatment agents in the nail involvement. Syuto et al. [47] have reported that low-dose systemic cyclosporine successfully treats nail psoriasis with an improvement rate of over 90% of the patients. The initial dose was 3 mg/kg/day twice a day and was reduced to 1.5 mg/kg/day in a single administration when the improvement was observed. Oral cyclosporine (3.5–4.5 mg/kg/day) in combination with topical calcipotriol cream (50 *μ*g/kg/day) has been shown to be more effective with a low risk of relapse in nail psoriasis when compared to the results of the patients receiving cyclosporine alone [48]. In a study by Mahrle et al. [49], 210 patients were randomly assigned for oral cyclosporine (2.5–5 mg/kg/day) or etretinate (0.5–0.75 mg/kg/day) for 10 weeks, and a significant alleviation of nail involvement was shown in both groups. At the second phase of this study, either systemic therapies were discontinued and were switched to topical dithranol or cyclosporine was tapered for 12 weeks. The tapered cyclosporine group was reported to show a significant improvement in nail involvement.

Gümüşel et al. [45] compared the efficacy and safety of methotrexate and cyclosporine in psoriatic nails of 34 patients in a randomized blinded study using NAPSI scores as an objective evaluation of the treatment outcomes. Initial methotrexate dose was 15 mg/week subcutaneously and was decreased after 3 months of treatment. The initial dose of cyclosporine was 5 mg/kg/day and was tapered to 2.5–3.5 mg/kg/day. After 6 months of treatment, they concluded that the two medications were similarly and moderately effective in nail psoriasis. Methotrexate was found to be more effective on nail matrix lesions, whereas cyclosporine was more effective in nail bed involvement.

In the retrospective study of Sánchez-Regeña et al. [44] comparing the classical and biological therapies in the treatment of nail psoriasis, all the classical and biological systemic treatments including cyclosporine, acitretin, methotrexate, and PUVA were shown to significantly reduce the severity of nail psoriasis, with the exception of narrow-band UVB (NUVB). Among the classical treatments, the improvement of nail psoriasis was more prominent in patients who had received cyclosporine. However, the percentage of change in the NAPSI score was significantly greater with biological treatments.

## 7. Biologic Treatments

In the last decade, the introduction of biological agents used for the treatment of plaque psoriasis and psoriatic arthritis has brought a safe and highly effective treatment option for severe nail bed and matrix disease as well. Both antitumor necrosis-*α* (TNF-*α*) and T-cell-targeted therapies have been useful for refractory severe nail psoriasis [8]. While biological therapies have been shown to be effective in nail psoriasis, infliximab appears to be the most effective option with the strongest evidence [7, 51–54] (Table 3).

Infliximab is a chimeric, human-murine IgG1 monoclonal antibody against TNF-*α*, which acts by neutralizing its biological activity. It is administered intravenously, with a dose of 0.5 mg/kg at weeks 0, 2, 6 and repeated every 8 weeks. In the first study evaluating the efficacy of infliximab in nail psoriasis, 25 patients with plaque-type psoriasis or psoriatic arthritis achieved a 50% reduction in mean NAPSI scores at week 14, while at week 22, the mean NAPSI score was 0 [53]. In a 50-week RCT (randomized controlled trial) of infliximab, 240 of 305 patients with nail psoriasis in the treatment arm started to show clearance of the nail disease as early as in week 10, with complete clearance in 44.7% of the patients at week 50. The treatment group had 26.8% and 57.2% improvements in the NAPSI scores at weeks 10 and 24, respectively, while worsening was observed in the placebo group. On the other hand, when the placebo group was switched to infliximab therapy at week 24, they achieved NAPSI scores comparable to those obtained with the original treatment group [54]. Further assessment of the results showed that the reduction of NAPSI scores was sustained, following 1 year of continuous infliximab therapy [54, 70]. A retrospective analysis of 48 patients with nail involvement confirmed the efficacy of infliximab therapy in nail psoriasis, with rapid and persistent improvements. At week 14, more than 50% reduction was observed in the mean NAPSI scores in 85.4% of patients. Improvement of nail psoriasis continued between weeks 14 and 38, with further reductions in NAPSI scores [55]. Similarly, in another study of 18 patients with psoriasis and psoriatic arthritis, a significant improvement was noted in most of the patients following the third infusion of infliximab, with a reduction of mean NAPSI scores from 55.8 at baseline, to 29.8 at week 14. At week 38, an almost complete resolution of psoriatic nail involvement was demonstrated [56]. Response to infliximab therapy has also been reported to be effective in patients with severe nail psoriasis refractory to other systemic therapies [71]. 

Adalimumab is a recombinant human IgG1 monoclonal antibody against TNF-*α*. It is administered subcutaneously, at a dose of 40 mg every other week, usually preceded by an 80 mg loading dose. In a study of 21 patients treated with adalimumab for psoriasis or psoriatic arthritis, with concomitant nail disease, improvement was observed as early as at 12 weeks in both fingernails and toenails [59]. For the fingernails, there was almost a 50% reduction in mean NAPSI scores at week 12 and almost complete resolution at week 24. In another study evaluating the effectiveness of adalimumab for nail psoriasis in 259 patients with psoriatic arthritis in a 12-week study, the mean NAPSI score was reduced by 44% at week 24 [58]. In a RCT of palmoplantar psoriasis, 28 of 36 patients with nail involvement received adalimumab therapy for 16 weeks and showed a higher mean percentage of NAPSI improvement compared to placebo-treated group (50% versus 8%). At week 16, patients from the placebo group were switched to receive active treatment, and a mean improvement of 38% was reported in NAPSI at week 28 [57].

Etanercept is a soluble, human, TNF-*α* receptor fusion protein. It binds TNF-*α* with greater affinity than natural receptors, so that TNF-*α* becomes biologically inactive. Etanercept is administered subcutaneously, usually at a dose of 50 mg twice weekly for 3 months, followed by once weekly thereafter. In a prospective trial of patients with moderate to severe plaque psoriasis, 69 patients with nail psoriasis were treated with etanercept. One treatment arm received etanercept 50 mg once weekly for 24 weeks. The other treatment arm received etanercept 50 mg twice weekly for the first 12 weeks and once weekly for the following 12 weeks. There was a significant improvement in mean NAPSI scores at week 24 in both groups, with significant number of patients showing complete resolution. Both treatment regiments were found to be effective in nail psoriasis [60]. In the post hoc analysis of a RCT, in which the patients were randomized to receive either continuous (25 mg twice weekly for 54 weeks) or paused (50 mg twice weekly up to 12 weeks, followed by 25 mg twice weekly in case of relapse) etanercept therapy, 564 patients with nail involvement were evaluated. The mean NAPSI scores were improved by 28.9% at week 12 and by 51% at week 54, while complete resolution was observed in 30% of patients [61]. There are also case reports demonstrating the rapid and marked improvement of nail psoriasis with etanercept therapy [72–74].

Golimumab is a human monoclonal antibody against TNF-*α*. It is administered subcutaneously. In a RCT of golimumab for psoriatic arthritis, response to nail involvement in 287 patients was assessed as a secondary outcome. Patients in the treatment arm received 50 mg or 100 mg golimumab every 4 weeks through week 24. Significant improvements in nail symptoms were observed in golimumab-treated patients as early as at week 14 in 50 mg and 100 mg group, with a reduction in NAPSI scores of 25% and 43% by week 14 and of 33% and 54% by week 24, respectively [62].

Alefacept is a recombinant human fusion protein composed of lymphocyte function-associated antigen-3 (LFA-3) and Fc portion of human IgG. It binds to CD2 receptor on T cells and thereby blocks T-cell interactions with antigen-presenting cells. In addition, it triggers the apoptosis of memory T cells and thus decreases the number of pathogenic T cells and the inflammatory response. It is administered weekly either 15 mg intramuscularly or 7.5 mg intravenously, generally for 12 weeks. There are a few studies with small number of patients in the literature, evaluating the efficacy of alefacept in nail psoriasis. In a group of 6 patients with mild nail psoriasis, after 12 weeks of intramuscular etanercept treatment, 3 demonstrated 30% or greater improvement in NAPSI scores at week 18, while 1 remained unchanged and 2 worsened [65]. Similarly in a group of 8 patients, 5 of which having moderate to severe nail psoriasis, 3 patients showed improvement, 3 remained unchanged, and 2 worsened [63]. In another study of 15 patients with severe nail psoriasis, baseline NAPSI scores were reduced by 39% at week 24, after 12 weeks of intramuscular therapy [64, 75]. 

Ustekinumab is a human monoclonal antibody that binds to the shared p40 subunit of the cytokines interleukin (IL)-12 and IL-23 to inhibit their biologic activity, as a consequence reduces IL-17A and IL-17F, and thus blocks the differentiation and proliferation of T helper (T_H_)-1 and T_H_-17 populations. It is administered subcutaneously, at a dose of 45 mg (90 mg if patient weight >100 kg), usually at weeks 0, 4 and every 12 weeks thereafter. In a case report of nail psoriasis unresponsive to etanercept, complete improvement was noted after the second injection of 45 mg subcutaneous ustekinumab at week 8 [75]. Igarashi et al. [68] randomized 158 patients with plaque psoriasis to receive ustekinumab 45 or 90 mg at weeks 0, 4 and every 12 weeks or placebo with crossover to ustekinumab at week 12. Among 102 patients with nail psoriasis, the improvements in NAPSI scores were significant at week 12 and continued to increase from week 12 to 64, with reductions of 57% at 45 mg group and 68% at 90 mg group at week 64. In a study of 27 patients with moderate-to-severe plaque psoriasis with nail involvement, excellent response to ustekinumab was demonstrated. NAPSI scores were improved by 42.5% at week 16, by 86.3% at week 28, and by 100% at week 40 [66]. Ustekinumab was also reported to be effective in a case of paradoxical psoriasis with severe scalp and nail involvement, which developed after adalimumab therapy for psoriatic arthritis [76]. In a recently published case series of 13 patients, who had been previously treated with at least four biologics with disappointing results, the patients received ustekinumab either as 90 mg monotherapy or 45 mg in combination with either methotrexate or cyclosporine. The mean percentage of reduction of the NAPSI score was 31.8% at week 12. Two patients treated in combination with cyclosporine 100 mg b.i.d. had complete resolution of their nail psoriasis at week 12 [67]. After longer term safety and efficacy are proven, ustekinumab could become an effective option for treatment of nail psoriasis.

Briakinumab, like ustekinumab, is a monoclonal antibody against p40 subunit of cytokines IL-12 and IL-23. It is administered subcutaneously. In a RCT of 317 patients with psoriasis, patients were randomized to receive either briakinumab (200 mg at weeks 0 and 4, followed by 100 mg every 4 weeks, from week 8 through 48) or methotrexate (5–25 mg per week, from week 0 through 51). Although the primary end point of the study was to assess the improvement in PASI scores, one of the secondary efficacy end points included the change in the NAPSI scores of the target fingernail, which was significantly lower with the briakinumab group at weeks 24 and 52, as compared with the methotrexate group [69].

All biologic agents currently available for the treatment of plaque psoriasis seem to be efficient in treating severe nail psoriasis, although no particular agent is approved for this purpose [7]. In an expert panel, participants strongly agreed that full nail clearance is an achievable goal using biologic therapy in psoriatic patients with nail involvement [52]. However, long-term safety data regarding use of this class of therapy is still being explored, and therefore the possible risks of treatment should be carefully weighted [52]. Most of the studies evaluating the efficacy of biologic agents in nail psoriasis are preliminary [77]. Among these only infliximab and golimumab were evaluated in RCTs, and significant improvements in nail psoriasis were achieved when compared to placebo [62, 70]. Of note, in the phase II trial of ixekizumab, an anti-IL-17 monoclonal antibody for chronic plaque psoriasis, significant improvements in NAPSI scores were reported as early as at 2 weeks with an impressive safety profile [78].

## 8. Treatment of Nail Psoriasis in Children

Despite the common onset of psoriasis in the childhood, validated clinical data on the epidemiology and the treatment of pediatric nail psoriasis is scant. In a recent multicenter study conducted in US, 39.2% of 181 children with psoriasis were found to have nail involvement [79]. Similar results were reported from Kuwait (37.81%), although in more than half of the cases the changes, pitting being the most common, were so subtle that the children were not aware of them [80]. Nail psoriasis was found to be more common in boys, probably related to koebnerization [78, 79]. There are no studies on the efficacy and safety of various treatments for nail psoriasis in children. The absence of clinical data corresponds with a lack of licensure for the pediatric use of many available treatments [81]. 

Data was available for the treatment of nail psoriasis from an epidemiologic study of pediatric psoriasis from Turkey. Thirteen children with nail psoriasis were treated with potent topical steroid ointments for onycholysis and nail bed hyperkeratosis; however, the results were unsatisfactory [82]. In a pilot study of 4 children to determine the efficacy of intralesional triamcinolone acetonide in the treatment of nail pitting in children, following a single dose, the degree of pitting was reduced by a mean of 15% in the second month and 42% in the fourth month [83]. 

In one case report, solely of nail psoriasis in a 6-year-old girl, tazarotene 0.05% gel daily applied for 8 weeks was found to be effective, especially in nail bed hyperkeratosis [84]. A 13-year-old girl with a 3-year history of episodic pustular eruption of the right thumb and psoriasiform changes on the fingers of the right hand, with a clinical diagnosis of Acrodermatitis Continua of Hallopeau, responded well to topical application of 0.1 mg/mL indigo naturalis extract oil [85]. Indigo naturalis, a Chinese herbal remedy extracted from the leaves of indigo-bearing plants, has been demonstrated to have antipsoriatic effects. Another case report of Acrodermatitis Continua of Hallopeau, in a 2-year-old boy, revealing severe nail dystrophy of 19 nails, which was resistant to occluded clobetasol and pimecrolimus and oral acitretin and methotrexate, was reported to demonstrate an excellent response to the combined treatment of thalidomide (50 mg/day for 5 months) and broad band ultraviolet B comb (2 months) [86]. 

In pediatric nail psoriasis, a combination of calcipotriene (calcipotriol) and betamethasone dipropionate seems to be a rational and simple approach, as both substances have shown to be safe and efficient in children [87].

## 9. Conclusions

Since severe psoriatic nail disease can lead to functional or emotional impairment, even as a sole manifestation of psoriasis, treatment should be individualized for each patient. Topical approaches or laser treatments may be suitable for limited disease or may take part in combination therapies for more severe disease, while classical systemic agents or biologic treatments are preserved for severe cases with extensive cutaneous disease or psoriatic arthritis.

## Figures and Tables

**Table 1:** Topical therapies for treatment of nail psoriasis.

Author	Year	*n*	Intervention	Comparison	Treatment protocol	Results	LoE [16]
Nakamura et al. [17]	2012	15	Clobetasol propionate at concentrations 0.05%, 1%, and 8%	Placebo (coat nail lacquer)	Twice weekly, for 4 mos	51% improvement in treatment group (8% clobetasol more efficient)	N/A

Fischer-Levanchini et al. [18]	2012	6	0.1% tazarotene ointment	—	Once daily, under occlusion, for 6 mos	88% improvement in NAPSI scores at 6th mo	N/A

De Simone et al. [19]	2012	21	0.1% tazarotene ointment	No treatment to the other hand	Once daily, to the affected nails of a randomly selected hand, for 3 mos	Statistically significant improvements in the treated hands at week 12	N/A

Tzung et al. [20]	2008	40	0.005% calcipotriol + 0.05% betamethasone dipropionate	0.005% calcipotriol	Calcipotriol twice daily and calcipotriol + betamethasone once daily for 3 mos	Similar efficacy in both groups, significant reduction of NAPSI scores	B

Sánchez-Regaña et al. [21]	2008	15	8% clobetasol in nail lacquer and tacalcitol	—	Clobetasol once daily at weekends and tacalcitol at weekdays under occlusion, for 6 mos	78% reduction in NAPSI at 6 mos	N/A

Rigopoulos et al. [22]	2007	46	0.1% tazarotene cream	0.05% clobetasol propionate	Once daily under occlusion, for 3 mos	Similar efficacy in both groups, significant reduction of NAPSI scores	A2

Regaña et al. [23]	2005	10	8% clobetasol in nail lacquer	—	Once daily, for 3 weeks and twice weekly, for 9 mos	Reduction of all nail alterations within 1 mo	N/A

Cannavò et al. [24]	2003	16	70% CsA oral solution in maize oil	Maize oil	For 3 mos	Complete resolution or substantial improvement in CsA group	A2

Bianchi et al. [25]	2003	25	0.1% tazarotene gel	—	Once daily, for 3 mos	19/25 good clinical response	N/A

Rigopoulos et al. [26]	2002	62	Calcipotriol cream + clobetasol propionate	—	Calcipotriol once daily every weeknight and clobetasol once daily every weekend, for the first 6 mos and twice weekly clobetasol for the 2nd 6 mos	Reduction at subungual hyperkeratosis: 72.3% at 6 mos and 81.2% at 12 mos	N/A

Scher et al. [27]	2001	31	0.1% tazarotene gel	Vehicle gel	Once daily, for 6 mos	Significant improvement of onycholysis and pitting in tazarotene group	A2/B

de Jong et al. [28]	1999	57	1% 5-FU in permeation enhancer lotion (Belanyx)	Belanyx (urea and propylene glycol)	Once daily, for 3 mos	Significant improvements with both preparations	A2

Baran and Tosti [29]	1999	18	8% clobetasol nail lacquer	Placebo	Once daily in the first week, from 2nd week onwards 2-3 times weekly, for up to 9 mos	Clear improvement in 80%, complete resolution in 22% of patients in the treatment arm	B

Tosti et al. [30]	1998	58	Calcipotriol ointment	Betamethasone propionate + salicylic acid	Twice daily, for up to 5 months	Calcipotriol as effective as the combination of topical steroid and salicylic acid (49% versus 51% reduction of subungual hyperkeratosis in fingernails at 6 mos)	B

Yamamoto et al. [31]	1998	20	0.4–2% anthralin in petrolatum	—	Once daily, for 5 mos	Effective in 12/20 patients, particularly in onycholysis and subungual hyperkeratosis	N/A

Fredriksson [32]	1974	20	1% 5-FU solution	—	Twice daily, for up to 6 mos	Considerable improvement in 17/20 patients, 75% reduction of symptoms compared to baseline	N/A

*n*: number of patients.

mo: month.

N/A: not applicable.

NAPSI: nail psoriasis severity index.

CsA: cyclosporine.

5-FU: 5-fluorouracil.

LoE: level of evidence (A2: randomized, double-blind, controlled trial of good quality, B: randomized controlled trial of poor quality).

**Table 2:** Systemic therapies for treatment of nail psoriasis.

Author	Year	*n*	Intervention	Comparison	Treatment protocol	Results	LoE [16]
Sánchez-Regaña et al. [44]	2011	84	Classical treatment	Biological treatment	Classical: acitretin, MTX, CsA, PUVA, NUVB, REPUVA, RENUVB Biological: infliximab, efalizumab, etanercept, adalimumab, for up to 8 mos	Significant reductions in NAPSI scores with all antipsoriatics, except for NUVB; significantly greater with CsA and biological as infliximab and adalimumab at 3 and 6 mos	N/A

Gümüşel et al. [45]	2011	37	MTX 15 mg/week, sc	CsA 5 mg/kg, po	MTX decreased to 10 mg/week after 3 months, for total 6 mos; CsA decreased to 2.5–3.5 mg/kg/day after 3 mos, for total 6 mos	Similar efficacy in both groups: reduction in NAPSI scores: 43% in MTX group, 37% in CsA group	A2

Tosti et al. [46]	2009	36	Acitretin	—	0.2–0.3 mg/kg/day, for 6 mos	41% reduction in NAPSI scores	N/A

Syuto et al. [47]	2007	16	CsA-MEPC	—	3 mg/kg/day and reduced to 1.5 mg/kg/day in responders	Improvement in over 90% of patients	N/A

Feliciani et al. [48]	2004	54	CsA	CsA + calcipotriol cream	CsA 3.5 mg/kg/day, in both groups; calcipotriol cream twice daily, for 3 mos	79% improvement in combination group and 47% improvement in CsA alone	N/A

Mahrle et al. [49]	1995	210	CsA	Etretinate	Phase 1: randomly assigned for CsA (2.5–5 mg/kg/day) or etretinate (0.5–0.75 mg/kg/day) for 10 weeks Phase 2: etretinate group discontinued treatment and continued with topical dithranol; CsA group either tapered or discontinued and replaced with topical dithranol for 12 weeks	After phase 1: significant alleviation of nail involvement in both groups and after phase 2: statistically significant decrease in nail involvement for tapered cyclosporine group	B

*n*: number of patients.

NAPSI: nail psoriasis severity index.

N/A: not applicable.

sc: subcutaneous.

po: peroral.

MTX: methotrexate.

CsA: cyclosporine.

MEPC: microemulsion preconcentrate.

LoE: level of evidence (A2: randomized, double-blind, controlled trial of good quality, B: randomized controlled trial of poor quality).

**Table 3:** Biologic therapies for treatment of nail psoriasis.

Author	Year	*n*	Intervention	Comparison	Protocol	Results	LoE [16]
Fabroni et al. [55]	2011	48	Infliximab	—	5 mg/kg, iv infusion at weeks 0, 2, 6 and every 8 weeks through week 38	NAPSI-50 is achieved in 85% of patients at week 14, 96% at week 22, 98% at week 38; NAPSI-75 is achieved in 23% of patients at week 14, 65% at week 22, 81% at week 38; NAPSI-90 is achieved in 29% of patients at week 38	N/A

Rich et al. [54]	2008	305	Infliximab	Placebo	5 mg/kg, iv infusion at weeks 0, 2, 6 and every 8 weeks through week 46	26% and 57% improvements in NAPSI scores at weeks 10 and 24 and complete clearance of target nail in 45% of patients at 1 year	A2

Rigopoulos et al. [56]	2008	18	Infliximab	—	5 mg/kg iv infusion at weeks 0, 2, 6 and every 8 weeks through week 38	Significant decrease in NAPSI scores (56 at baseline to 30 at week 14, 16 at week 22, 7 at week 30, and 3.3 at week 38)	N/A

Bianchi et al. [53]	2005	25	Infliximab	—	5 mg/kg, iv, at weeks 0, 2, 6, 14, 22	NAPSI-50 is achieved in all patients at week 14; NAPSI-75 is achieved in all patients at week 22	N/A

Leonardi et al. [57]	2011	36	Adalimumab	Placebo	80 mg, sc at week 0, 40 mg every other week starting at week 1, through week 16; patients in the placebo group were started to receive active treatment starting at week 16, through week 28	Significantly higher improvement in NAPSI scores in the treatment arm (50% versus 8%) at week 16; once switched to adalimumab, patients in the initial placebo group improved 38% at week 28, while patients who began the study with adalimumab continued to improve to 54%	N/A

Van den Bosch et al. [58]	2010	259	Adalimumab	—	40 mg, sc, at every other week through week 12	Mean NAPSI scores are reduced by 44% at week 12	N/A

Rigopoulos et al. [59]	2010	21	Adalimumab	—	80 mg, sc at week 0, 40 mg every other week starting at week 1, through week 24	Significant improvement in all patients after 8th injection; fingernail NAPSI decreased from 11 at baseline to 4 at week 24 in patients with just cutaneous psoriasis and from 24 to 10 in patients with psoriatic arthritis	N/A

Ortonne et al. [60]	2012	69	Etanercept	Etanercept	1st group 50 mg weekly for 24 weeks and 2nd group 50 mg twice weekly for the first 12 weeks, 50 mg weekly for the other 12 weeks, sc	Both dose regimens are effective for nail psoriasis and significant improvement in NAPSI scores in both groups at week 24	N/A

Luger et al. [61]	2009	564	Etanercept	—	25 mg twice weekly for 54 weeks or 50 mg twice weekly for 12 weeks, continued with 25 mg twice weekly in case of relapse, sc	NAPSI scores improved by 29% at week 12, by 51% at week 54, complete resolution in 30% of patients	N/A

Kavanaugh et al. [62]	2009	287	Golimumab	Placebo	50 or 100 mg, sc, every 4 weeks through week 24	Significant improvements started as early as at week 14: 25% reduction in NAPSI scores in 50 mg group, 43% reduction in 100 mg group at week 14, 33% reduction in 50 mg, and 54% reduction in 100 mg group at week 24	B

Körver et al. [63]	2006	8	Alefacept	—	15 mg weekly, im, for 12 weeks	3 patients showed significant improvement, 3 patients unchanged, and 2 patients worsened	N/A

Parrish et al. [64]	2006	15	Alefacept	—	15 mg weekly, im, for 12 weeks	39% reduction in NAPSI scores at week 24	N/A

Cassetty et al. [65]	2005	6	Alefacept	—	15 mg weekly, im, for 12 weeks	3 patients showed ≥30% improvement in NAPSI scores, 1 unchanged, and 2 worsened	N/A

Patsatsi et al. [66]	2013	27	Ustekinumab	—	45 mg, sc, at weeks 0, 4 and every 12 weeks thereafter (90 mg if patient weight > 100 kg)	Significant improvements in NAPSI scores (43% at week 16, 86% at week 28, and 100% at week 40)	N/A

Vitiello et al. [67]	2013	13	Ustekinumab	—	90 mg (*n* = 5, patients weight > 100 kg), sc, at weeks 0, 4 and every 12 weeks thereafter or 45 mg in combination with MTX (*n* = 6) or CsA (*n* = 2)	38% reduction in NAPSI scores in monotherapy group, 27% reduction in MTX combination, complete resolution in CsA combination group, at week 12	N/A

Igarashi et al. [68]	2012	102	Ustekinumab	Placebo	45 or 90 mg, sc, at weeks 0, 4 and every 12 weeks through 72 weeks, placebo group with crossover to ustekinumab at week 12	Improvement in NAPSI scores: 57% in 45 mg group, 68% in 90 mg group at week 64	A2

Reich et al. [69]	2011	317	Briakinumab	MTX	Briakinumab 200 mg at weeks 0 and 4, 100 mg every 4 weeks through week 48, sc, MTX 5–25 mg/week for 51 weeks	NAPSI scores of the target fingernail significantly lower with the briakinumab group at weeks 24 and 52, as compared with the methotrexate group	N/A

*n*: number of patients.

sc: subcutaneous.

iv: intravenous.

im: intramuscular.

N/A: not applicable.

NAPSI: nail psoriasis severity index.

MTX: methotrexate.

CsA: cyclosporine.

LoE: level of evidence (A2: randomized, double-blind, controlled trial of good quality, B: randomized controlled trial of poor quality).
